# Subjective smell and taste dysfunctions and prognosis in patients with COVID-19 admitted to a major public hospital in Southern Brazil: A retrospective cohort study (NUTRICOVID19)

**DOI:** 10.1097/MD.0000000000041866

**Published:** 2025-03-21

**Authors:** Caio Wolff Ramos Baumstein, Vivian Cristine Luft, Caroline Marques de Lima Cunha, Zilda Elizabeth de Albuquerque Santos, Valesca Dall’Alba, Anderson Garcez, Raquel Canuto, Maria Teresa Anselmo Olinto

**Affiliations:** a Post-graduate Program in Food, Nutrition and Health, Department of Nutrition, Faculty of Medicine, Federal University of Rio Grande do Sul, UFRGS, Porto Alegre, Rio Grande do Sul, Brazil; b Nutrition Service, Hospital de Clínicas de Porto Alegre, Federal University of Rio Grande do Sul, UFRGS, Porto Alegre, Rio Grande do Sul, Brazil; c Department of Nutrition, Faculty of Medicine, Federal University of Rio Grande do Sul, UFRGS, Porto Alegre, Rio Grande do Sul, Brazil; d Post-graduate Program in Medical Sciences: Endocrinology, Department of Internal Medicine, Faculty of Medicine, Federal University of Rio Grande do Sul, UFRGS, Porto Alegre, Rio Grande do Sul, Brazil.

**Keywords:** anosmia, COVID-19, dysgeusia, mortality, prognosis

## Abstract

The literature notes that coronavirus disease 2019 (COVID-19) patients with olfactory disturbances tend to have lower disease severity and that olfactory disturbances may act as a protective factor against mortality. So, this study aimed to explore the association of smell and/or taste disturbance with disease severity and all-cause mortality among patients who were hospitalized with COVID-19. A retrospective cohort study (Nutrition and COVID-19 Study [NUTRICOVID19]) was conducted with 1331 patients (men and women, age ≥ 18 years) hospitalized with COVID-19 between June and December 2020. Poisson and Cox regressions were used to investigate the unadjusted and adjusted associations between smell and/or taste disturbance and the following prognostic indicators: length of stay (LOS), intensive care unit admission, need for invasive mechanical ventilation (IMV), and mortality. Patients with altered smell and/or taste had a shorter LOS (9 vs 11 days), were less likely to require IMV (22% vs 35.1%), and experienced lower mortality (17.1% vs 29.2%) compared to those without such symptoms. After multivariable adjustment, patients with smell and/or taste disturbances were 33% less likely to need IMV compared to those without such symptoms (RR = 0.67; 95% CI = 0.50–0.88), but the relationship between these symptoms and mortality lost statistical significance. In this population of patients with COVID-19, the presence of smell and/or taste disturbances was associated to lower rates of IMV.

## 1. Introduction

Coronavirus disease 2019 (COVID-19), an acute respiratory infection caused by the severe acute respiratory syndrome coronavirus 2 (SARS-CoV-2), spreads primarily from person to person.^[1]^ In December 2019, the first cases of the disease were identified in China; it soon spread to Europe, the United States and, later, to the entire world. In March 2020, COVID-19 was declared a pandemic by the World Health Organization.^[2]^ By February 2023, it is estimated that the novel coronavirus has infected more than 754 million individuals and caused more than 6.8 million deaths worldwide.^[3]^ In Brazil, estimates account for approximately 36 million cases of COVID-19 in this period;^[4]^ however, consistent with the continental dimensions and high economic disparity of the country, there was great variability in the distribution of cases and deaths. In 2020, for example, a comparison of the mortality burden in 2 state capitals in extreme opposites of the country – Fortaleza, in the Northeastern state of Ceará, and Porto Alegre, in the Southern state of Rio Grande do Sul – revealed rates of 42 deaths versus 5.4 deaths per million people, respectively.^[5]^

COVID-19 presents on a spectrum of severity that ranges from asymptomatic infection to life-threatening disease.^[1,6,7]^ The main symptoms reported by those infected are cough and fever.^[7]^ Myalgia, fatigue, diarrhea, nausea, anorexia, headache, anosmia (loss of smell) and dysgeusia (taste dysfunction) are also frequent.^[8]^ In 2022, a systematic review with meta-analysis which included 17,452 patients across 26 studies, identified an overall prevalence of anosmia and dysgeusia of 56% (95% CI = 41% to 71%).^[9]^ However, there was significant variability between studies, ranging from 6% (95% CI = 3% to 9%) to 99% (95% CI = 99% to 100%). The prevalence also varies according to demographic characteristics, being highest in younger and female patients.^[10,11]^ Conversely, in a meta-analysis of 13,813 patients across 26 studies detected no such difference in prevalence by gender and found a higher prevalence of smell and taste disturbance in older patients.^[12]^

Due to the differences in the onset and timing of these symptoms across studies, some authors have suggested that the presence of smell and/or taste disturbances be used as criteria for the clinical diagnosis of COVID-19 and as a tool to estimate the prognosis of affected individuals.^[13–15]^ Regarding prognosis, the literature notes that patients with olfactory disturbances tend to have lower disease severity^[16]^ and that olfactory disturbances may act as a protective factor against mortality.^[17]^ Corroborating these findings, a 2022 meta-analysis showed less need for hospitalization, less need for intubation and/or IMV, and lower mortality among patients with COVID-19 who experienced smell and/or taste disturbances.^[18]^ Finally, it is now well known that the presence of comorbidities can alter the development of smell and/or taste disturbances. Some studies have found a negative association between the presence of comorbidities such as cardiovascular diseases (CVD), hypertension, and diabetes mellitus with changes in smell and/or taste.^[10,13,19]^ In others, however, no such association was observed.^[12,20]^

Within this context, the present study sought to contribute with knowledge regarding the relationship between smell and/or taste disturbances and disease prognosis (severity and mortality) of patients hospitalized for COVID-19 during the first year of the pandemic in Brazil. Secondarily, we sought to explore the relationship of sociodemographic characteristics and comorbidities with these symptoms.

## 2. Methods

### 2.1. Study design

Retrospective cohort study of data obtained from electronic medical records of patients admitted to a public, university-affiliated tertiary referral center.

### 2.2. Population, sample, and sampling

The study population consisted of patients hospitalized for COVID-19 at Hospital de Clínicas de Porto Alegre (HCPA), Rio Grande do Sul, Brazil, between June 1, 2020, and December 31, 2020. This study is nested within a larger project, “Nutrition and COVID-19 Study (NUTRICOVID19) – Aspectos nutricionais e sua associação com o prognóstico de pacientes hospitalizados por COVID-19 em Porto Alegre/RS” [Nutritional aspects and their association with the prognosis of patients hospitalized for COVID-19 in Porto Alegre, Rio Grande do Sul], which was approved by the HCPA Research Ethics Committee (IRB-equivalent) under number 20200388. During the pandemic, HCPA was designated as a statewide referral hospital for moderate and severe cases of COVID-19. This retrospective chart review study involving human participants was in accordance with the ethical standards of the institutional and national research committee and with the 1964 Helsinki Declaration and its later amendments or comparable ethical standards. Informed consent was obtained from all patients.

Sampling of patient records was done sequentially. All patients aged 18 years or older, regardless of sex, who tested positive for COVID-19 by RT-PCR at the time of admission and had a record of hospitalization at any HCPA inpatient unit or intensive care unit (ICU) were included (n = 1575). The exclusion criteria were pregnancy, breastfeeding, length of hospital stay (LOS) < 24 hours, withdrawal from treatment and/or hospitalization, transfer to outside hospitals during hospitalization, and lack of data on smell and/or taste disturbance. The final sample size included 1331 patients.

The statistical power to test for association between smell and/or taste disturbances (exposure) and the outcomes of interest (disease severity and mortality) was calculated *a posteriori*. Thus, at a 95% confidence level, an unexposed/exposed ratio of 0.15, with rates of the outcomes of interest ranging from 17.1% to 76.4% in the unexposed population and a risk ratio ranging from 0.97 to 0.35, the statistical power of the sample was 8.4%, 27%, 89.6%, and 93.1%, respectively, for the association with supplemental oxygen therapy, ICU admission, Invasive Mechanical Ventilation (IMV), and mortality. This calculation was performed in the online version of the PSS Health tool.^[21]^

### 2.3. Data collection and study instruments

Information was collected by reviewing patients’ electronic medical records. Due to pandemic-related restrictions, all research activities were carried out remotely (online). A standardized questionnaire designed to collect information on sociodemographic characteristics and preexisting comorbidities, aiming to describe the sample and potential confounding factors, was prepared in Google Forms. This form was also used to record information on the patients’ prognosis and on the presence of smell and/or taste disturbances. A detailed instruction manual for collection of each item in the questionnaire was also prepared. The data collection team included undergraduate and graduate students in the health field. All received online training which included an introduction to the questionnaire and instruction manual, followed by standardization of data collection logistics. A self-explanatory video on collection and entry of data into the questionnaire was also created and made available. Training was also carried out during data collection, in order to maintain the internal validity of the study. Data collection from medical records took place between October 2020 and July 2022, and each patient was included in the study only after the end of their hospital stay, i.e., once their status regarding the mortality endpoint (alive or dead) was already established.

### 2.4. Primary variable (smell and/or taste disturbance)

The primary variable of this study was “subjective smell and taste dysfunctions and prognosis in adult patients with COVID-19 admitted to a major public hospital in Southern Brazil smell and/or taste disturbance.” The term “disturbance” includes reduction, distortion, or complete loss of each of the 2 senses (smell and taste). Therefore, for the purposes of this study, “smell and/or taste disturbance” encompasses hyposmia (reduced olfaction), anosmia (complete loss of olfaction), parosmia (distortion of perceived odors), hypogeusia (reduced gustation), ageusia (complete loss of gustation), and dysgeusia (distortion of perceived tastes). Information on the presence of these symptoms was collected at the time of hospital admission during the initial history and entered into the medical record, from which it was collected into the aforementioned questionnaire. The presence of smell and/or taste disturbances was recorded as follows: altered olfaction only; altered gustation only; both olfaction and gustation altered. This was subsequently categorized into a dichotomous variable, i.e., presence of smell and/or taste disturbance (0 – no; 1 – yes).

### 2.5. Prognostic variables

Variables related to disease severity during hospitalization were used for prognostic evaluation, in addition to a variable concerning vital status at the end of the hospital stay.

The severity variables were: need for supplemental oxygen therapy (yes/no), need for ICU admission (yes/no), length of ICU stay (continuous variable in days), need for IMV (yes/no), duration of IMV (continuous variable in days), and LOS. LOS was defined as the period calculated between the date of admission and the date of hospital discharge or death, regardless of each patient’s journey within the hospital.

Vital status: defined according to the mortality outcome at the end of hospitalization, i.e., alive (discharged) or dead. In the present study, the term “mortality” was also used for this variable, since deaths may or may not have been due to COVID-19 (and, therefore, would not constitute case fatality).

### 2.6. Covariables

Sociodemographic variables and information on comorbidities were collected to characterize the sample and control for confounding factors, namely:

- Sociodemographic variables: sex (male/female), age (continuous and dichotomous variable, <60 years old/≥ 60 years old), skin color/race (white/other), educational attainment (elementary or less/some high school/high school graduate/any higher), and place of residence (Porto Alegre/other cities in the state of Rio Grande do Sul);

- Comorbidities: known history of hypertension, obesity, diabetes mellitus, cardiovascular disease, chronic kidney disease, current or past malignancy, psychiatric disorders, chronic lung disease, endocrine disease, neurological disease, rheumatic disease, gastrointestinal disease, chronic liver disease, or allergies; presence of these comorbidities was assessed dichotomously (yes/no) at the time of hospital admission.

### 2.7. Statistical analyses

All analyses were performed in SPSS Version 18.0.3. For descriptive analysis, medians and interquartile ranges (IQR) were calculated for continuous variables, and absolute and relative frequencies for categorical variables; for smell and/or taste disturbance, the respective 95% confidence intervals (95% CI) were calculated. On bivariate analysis, the chi-square test with Pearson *P*-value for heterogeneity of proportions was used for dichotomous and nominal categorical variables, while the *P*-value for linear trend was used for ordinal variables. For the selection of potential confounding factors in the multivariate models, a *P*-value < .1 was used as the cutoff point. Unadjusted and adjusted Poisson regression models were used to test for an independent association between smell and/or taste disturbances and need for supplemental oxygen therapy, need for ICU admission, and need for IMV. Cox proportional hazards regression models, again both unadjusted and adjusted, were used to test for an independent association between smell and/or taste disturbances and mortality. For the Cox regressions, the date of onset of symptoms was used as time 0. In the absence of this data point, the date of patient admission was used instead; discharge or death dates were used to define the censoring time or correct outcome. For all analyses, we considered *P* < .05 as statistically significant.

## 3. Results

The final sample of this study consisted of 1331 patients with a median age of 62 (IQR = 49–71) years; 52.4% were male, 81.7% were white, the majority (55%) had not graduated high school, and 60% lived in the state capital, Porto Alegre. The most prevalent comorbidities among patients hospitalized for COVID-19 were high blood pressure (56.9%), obesity (37.2%), diabetes mellitus (34%), and other cardiovascular diseases (29.5%) (Table 1).

**Table 1:** Sample distribution and prevalence of smell and/or taste disturbances, stratified by sociodemographic characteristics and pre-existing comorbidities, in patients hospitalized for COVID-19 at a public hospital in southern Brazil. Porto Alegre, Rio Grande do Sul, June to December 2020 (n = 1331).

Variable	Sample distribution, n (%)	No smell and/or taste disturbance, n (%)	Smell and/or taste disturbance, n (%)	*P*-value
Total	1331 (100)	1156 (86.9)	175 (13.2)	–
Sex				
Male	697 (52.4)	609 (87.4)	88 (12.6)	.554
Female	634 (47.6)	547 (86.3)	87 (13.7)	
Age (yr)				
<60	596 (47.8)	496 (83.2)	100 (16.8)	<.001
≥60	735 (52.2)	660 (89.8)	75 (10.2)	
Ethnicity/skin color				
White	1086 (81.7)	945 (87)	141 (13)	.657
Other*	242 (18.3)	208 (86)	34 (14)	
Educational attainment				
Low educational level†	28 (2.1)	26 (92.9)	2 (7.1)	.317
Incomplete high school	714 (53.6)	622 (87.1)	92 (12.9)	
Complete high school	318 (23.9)	271 (85.2)	47 (14.8)	
Any higher education	142 (10.7)	117 (82.4)	25 (17.6)	
Not reported	129 (9.7)	–	–	
City of residence				
Porto Alegre	800 (60.2)	680 (85.0)	120 (15.0)	.016
Other‡	531 (39.8)	476 (89.6)	15 (10.4)	
Presence of comorbidities				
Hypertension				
Yes	757 (56.9)	667 (88.1)	90 (11.9)	.119
Obesity				
Yes	495 (37.2)	416 (84)	79 (16)	.020
Diabetes mellitus				
Yes	452 (34)	399 (88.3)	53 (11.7)	.271
Cardiovascular disease				
Yes	392 (29.5)	358 (91.3)	34 (8.7)	.002
Chronic kidney disease				
Yes	208 (15.6)	190 (91.3)	18 (8.7)	.037
Psychiatric/mental disorders				
Yes	188 (14.1)	129 (81.6)	28 (18.4)	.039
Current or past malignancy				
Yes	187 (14)	174 (93)	13 (7)	.007
Chronic lung disease				
Yes	157 (11.8)	140 (89.2)	17 (10.8)	.360
Endocrine disease				
Yes	110 (8.3)	97 (88.2)	13 (11.8)	.667
Neurological disease				
Yes	100 (7.5)	90 (90)	10 (10)	.333
Rheumatic/joint diseases				
Yes	93 (7)	76 (81.7)	17 (18.3)	.129
Gastrointestinal diseases				
Yes	87 (6.5)	77 (88.5)	10 (11.5)	.637
Chronic liver disease				
Yes	69 (5.2)	65 (94.2)	4 (5.8)	.063
Allergy				
Yes	52 (3.9)	47 (90.4)	5 (9.6)	.442

* Brown, black, yellow, indigenous.

† Incomplete primary education.

‡ Any other municipality in the state of Rio Grande do Sul.

**Figure 1.**
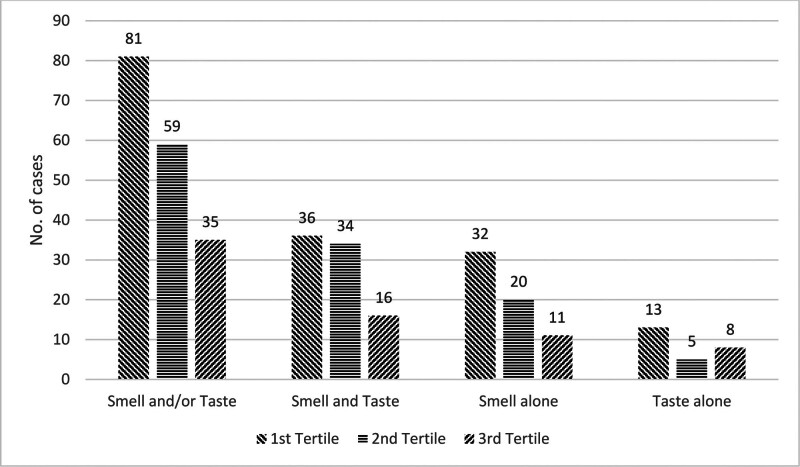
Comparison of prevalence of smell and/or taste disturbance across the study period (divided into tertiles).

Regarding smell and/or taste disturbances, 13.2% (95% CI = 11.3% to 14.9%) of the sample had at least 1 such symptom, with 149 (11.2%) and 112 (8.5%) patients reporting altered smell and taste, respectively. Figure 1 shows the prevalence of these symptoms over the study period. There was a gradual reduction in the number of patients reporting smell and/or taste disturbances between June 1, 2020, and late December 2020.

The presence of smell and/or taste disturbances was more prevalent in patients < 60 years of age and residents of the city of Porto Alegre, but there was no difference between men and women. Furthermore, these symptoms were more frequent in patients with psychiatric disorders and those with obesity.

Regarding disease prognosis, Table 2 shows that patients with smell and/or taste disturbance had a shorter median LOS (9 vs 11 days), and a smaller proportion of these patients required IMV (22.9 vs 35.1%) when compared to patients without such symptoms. However, among those who did require IMV, patients *without* smell and/or taste disturbances had a shorter ventilation time (13 vs 21 days on IMV) compared to those *with* smell and/or taste disturbances. Finally, regarding vital status at hospital discharge, patients who reported smell and/or taste disturbances had a lower mortality rate than those who did not report these symptoms on admission (17.1% vs 29.2%, respectively). The cumulative incidence of deaths (mortality) across the total sample of 1331 patients was 27.6%.

**Table 2:** Association of smell disturbance (hyposmia/anosmia) and/or taste disturbance (dysgeusia/ageusia) with disease severity and mortality in patients hospitalized for COVID-19 at a public hospital in southern Brazil. Porto Alegre, Rio Grande do Sul, June to December 2020 (n = 1331).

Hospitalization and prognosis factors	Smell and/or taste disturbance		*P*-value
	NO Median (IQR)/n (%)	YES Median (IQR)/n (%)	
LOS (d)	11 (6–22)	9 (5–17)	.009
Supplemental oxygen required			
No	272 (23.6)	37 (21.1)	.486
Yes	884 (76.4)	138 (78.9)	
ICU admission required			
No	607 (52.5)	102 (58.3)	.153
Yes	625 (47.5)	73 (41.7)	
Length of ICU stay (d)	12 (6–22)	9 (4–25)	.462
IMV required			
No	750 (64.9)	135 (77.1)	.001
Yes	406 (35.1)	40 (22.9)	
Duration of IMV (d)	13 (6–23)	21 (12–28)	.005
Vital status at discharge			
Alive	818 (70.8)	145 (82.9)	.001
Dead	333 (29.2)	30 (17.1)	

ICU = intensive care unit, IMV = invasive mechanical ventilation, IQR = interquartile ranges, LOS = length of stay.

Table 3 shows the unadjusted and adjusted (multivariate) analyses of the relationship between presence of smell and/or taste disturbances and disease severity and mortality variables. The association with need for supplemental oxygen therapy and length of ICU stay were not statistically significant on multivariate analyses. Conversely, the relationship between smell and/or taste disturbance and need for IMV remained statistically significant in all 3 adjusted models; after adjusting for sex and comorbidities, it was associated with a 33% reduction (95% CI = 0.50–0.88, *P* = .005) in need for IMV. Regarding mortality, the unadjusted model showed a 35% lower risk of death among patients with smell and/or taste disturbances, but this association lost its statistical significance after adjustment.

**Table 3:** Unadjusted and adjusted models for analysis of smell disturbance (hyposmia/anosmia) and/or taste disturbance (dysgeusia/ageusia) according to need for supplemental oxygen, need for ICU admission, need for mechanical ventilation, and mortality in patients hospitalized for COVID-19 at a public hospital in southern Brazil. Porto Alegre, Rio Grande do Sul, June to December 2020 (n = 1331).

Models	Supplemental oxygen required		
	PR	95% CI	*P*-value
Unadjusted model	1.03	0.95 to 1.12	.469
Adjusted model*	1.05	0.96 to 1.14	.258
Adjusted model†	1.05	0.97 to 1.14	.242
Adjusted model‡	1.05	0.97 to 1.14	.255
	ICU admission required		
	PR	95% CI	*P*-value
Unadjusted model	0.88	0.73 to 1.06	.170
Adjusted model§	0.89	0.73 to 1.06	.180
Adjusted model∥	0.89	0.74 to 1.06	.195
Adjusted model¶	0.90	0.75 to 1.08	.265
	Invasive mechanical ventilation		
	PR	95% CI	*P*-value
Unadjusted model	0.35	0.32 to 0.38	.003
Adjusted model#	0.38	0.34 to 0.42	.003
Adjusted model**	0.66	0.50 to 0.88	.004
Adjusted model††	0.67	0.50 to 0.88	.005
	Death		
	HR	95% CI	*P*-value
Unadjusted model	0.65	0.45 to 0.95	.026
Adjusted model‡‡	0.73	0.50 to 1.06	.102
Adjusted model§§	0.74	0.51 to 1.08	.122
Adjusted model∥∥	0.74	0.51 to 1.08	.114

HR = hazard ratio, ICU = intensive care unit, PR = prevalence ratio.

* Adjusted for age.

† Adjusted for age and 1 or more comorbidities (dichotomous variable).

‡ Adjusted for age and comorbidities (continuous variable).

§ Adjusted for sex.

∥ Adjusted for sex and 1 or more comorbidities (dichotomous variable)

¶ Adjusted for sex and comorbidities (continuous variable).

# Adjusted for sex.

** Adjusted for sex and 1 or more comorbidities (dichotomous variable).

†† Adjusted for sex and comorbidities (continuous variable).

‡‡ Adjusted for age.

§§ Adjusted for age and 1 or more comorbidities (dichotomous variable).

∥∥ Adjusted for age and comorbidities (continuous variable).

## 4. Discussion

This retrospective cohort study of 1331 patients hospitalized with COVID-19 demonstrated that the presence of altered smell and/or taste was associated to lower rates of IMV. Furthermore, patients with these symptoms had a shorter overall LOS and lower mortality when compared to patients without smell and/or taste disturbance.

Our findings suggest that patients with altered smell and/or taste were 33% less likely to need IMV than those without such symptoms, even after multivariable adjustment. Corroborating these findings, a prospective observational study carried out in 3 Brazilian public hospitals showed 69% lower odds of IMV in patients with altered taste and smell (OR = 0.311, 95% CI = 0.105–0.921, *P* = .027).^[22]^ In addition, a systematic review with meta-analysis using data from 8 studies (3823 patients) found a sevenfold greater need for IMV in patients without smell disturbances when compared to those who presented with these symptoms.^[18]^

It also bears stressing that, although patients with smell and/or taste disturbance were less likely to need IMV, paradoxically, among those who needed ventilation, those with altered smell/taste had a longer duration of IMV than those without these symptoms. This finding partially contradicts 1 of our hypotheses: that the presence of smell/taste disturbances, in addition to being a factor associated to lower rates of IMV, could reduce the duration of IMV.

Our study found a shorter LOS – and prior work found a lower hospitalization rate – for patients with these symptoms when compared to those without.^[13,18]^ In addition, this finding points towards 1 of our initial hypotheses, i.e., suggesting that altered smell and/or taste could be a protective factor of severity and, therefore, that the presence of this symptom could lead to both a lower need for hospitalization and a shorter LOS among those who are ultimately hospitalized.

The cumulative incidence of mortality in our study was 27.6% for all hospitalized patients, rising to 47.4% when only patients who required ICU admission were taken into account. These rates are similar to those described in a cohort study that monitored hospitalized patients admitted between March and September 2020 and found an in-hospital mortality of 22% overall and 47.6% among patients admitted to ICUs.^[23]^ It is important to note that, in both studies, these are all-cause mortality rates, not COVID-19 case fatality rates.

Regarding the association between vital status at discharge and smell/taste disturbance, we found a lower mortality in patients with these symptoms on unadjusted analysis (HR = 0.65, 95% CI = 0.45–0.95, *P* = .026); on the other hand, this relationship was no longer statistically significant in any of the 3 multivariate models. This finding disagrees with the results of 2 previous studies, which reported 37% lower odds of death (OR = 0.63, 95% CI = 0.39–0.95) and 82% lower odds of death (OR = 0.18, 95% CI = 0.07–0.47), respectively, in their multivariate analyses, as well as those of a meta-analysis which, using data from 6 studies (n = 4026), found a sevenfold higher mortality rate in patients without smell and taste disturbances.^[18,19,24]^

One hypothesis raised regarding the positive prognosis of patients who experience altered smell and taste is the fact that a more advanced local immunity would account for a more intense response to the infection, on the 1 hand generating more pronounced ear, nose, and throat symptoms while, on the other, leading to only mild respiratory symptoms.^[25]^ Another possibility discussed in the literature is that patients who present with these disturbances as their initial symptoms of COVID-19 may seek medical attention earlier, which may account for the shorter LOS among those with altered smell and/or taste (9 vs 11 days in the present sample). This is consistent with a prospective cohort study that described a weak negative correlation between presence of these symptoms and LOS (ρ = −0.139, *P* = .03).^[22]^ Such data corroborate the findings that patients with smell and taste alterations experience a faster recovery and, consequently, a better prognosis.^[18]^

We identified a lower presence of smell and/or taste disturbances than described in other studies published recently. A prospective study including hospitalized patients who tested positive for COVID-19 in northern Taiwan reported a 35.9% prevalence of smell and/or taste dysfunction among its population^[26]^ versus the 13.2% found in our sample. As for altered olfaction, 2 cross-sectional studies reported prevalences of 66.3% and 75.5%, respectively;^[27,28]^ in our study, this rate was only 11.2%. A low prevalence of impaired olfaction (only 5.1% of patients) was also obtained in a retrospective case series published in 2020.^[29]^ Regarding taste dysfunction, a 35.9% prevalence of dysgeusia was described in hospitalized patients with a positive RT-PCR test for COVID-19,^[30]^ compared to 8.5% in the present study. One possible explanation for these differences is heterogeneity in methods. The aforementioned studies used specific/validated tools or questionnaires to assess smell and/or taste involvement, a methodology that cannot be applied to retrospective studies. Furthermore, with the emergence of new variants of SARS-CoV-2, the prevalence of certain symptoms can vary significantly; the Omicron variant is known to be associated with a much lower rate of smell and taste disturbance compared to previous subvariants, such as Alpha and Gamma,^[31]^ which may be another explanation for the difference between prevalence of symptoms found across studies.

When analyzing the relationship between loss of smell and/or taste and the demographic characteristics and comorbidities of hospitalized patients, a significantly higher number of cases of smell and/or taste disturbance was found in patients aged < 60 years, which is consistent with studies previously published.^[13,32]^ Finally, we also found a higher prevalence of these symptoms in patients from Porto Alegre when compared to patients from other cities in the state of Rio Grande do Sul.

The cohort design of this study provides scientific evidence of reasonably high quality for the assessment of a causal relationship between the investigated exposure and the outcomes of interest. In addition, although the data refer only to the first year of the COVID-19 pandemic, we excluded the very earliest phase during which health services and hospitals were still adapting and reorganizing to establish new care routines and protocols in response to an unusual situation. Therefore, at the start of our study, routine care for COVID-19 cases was already well established at our facility (the largest referral hospital in the state). The indirect manner in which data on smell and taste disturbances were originally collected (i.e., reported by patients during history-taking, considering subjective evaluation of smell and taste function) suggests that the actual number of cases may be much higher than that recorded in this study.^[33]^ If true, such underreporting may have reduced the statistical power of the sample, but this would not affect the direction of the investigated associations. Notably, our sample size had low statistical power to test for association with need for supplemental oxygen therapy and ICU admission. For mortality, the statistical power was sufficient to test for association on unadjusted analysis, but it did not survive multivariable modeling to control for potential confounding factors. As for the association with IMV, the sample size had sufficient statistical power to demonstrate an association even after adjustments in the multivariate models. It also bears stressing that this study considered all-cause mortality, not case fatality, in patients hospitalized with COVID-19; deaths were not necessarily due to COVID-19 itself.

## 5. Conclusion

This study adds to the body of evidence on the most likely course of COVID-19 in patients experiencing smell and/or taste disturbances (shorter hospital stay and lower likelihood of IMV). More studies are needed to elucidate the pathophysiological mechanisms involved in the relationship between smell/taste disturbances and disease severity and mortality in COVID-19.

## Author contributions

**Conceptualization:** Caio Wolff Ramos Baumstein, Vivian Cristine Luft, Caroline Marques de Lima Cunha, Zilda Elizabeth de Albuquerque Santos, Valesca Dall’Alba, Anderson Garcez, Raquel Canuto, Maria Teresa Anselmo Olinto.

**Formal analysis:** Caio Wolff Ramos Baumstein, Maria Teresa Anselmo Olinto.

**Investigation:** Caio Wolff Ramos Baumstein, Vivian Cristine Luft, Zilda Elizabeth de Albuquerque Santos, Valesca Dall’Alba, Raquel Canuto, Maria Teresa Anselmo Olinto.

**Methodology:** Caio Wolff Ramos Baumstein, Caroline Marques de Lima Cunha, Anderson Garcez, Raquel Canuto, Maria Teresa Anselmo Olinto.

**Writing – original draft:** Caio Wolff Ramos Baumstein, Vivian Cristine Luft, Caroline Marques de Lima Cunha, Zilda Elizabeth de Albuquerque Santos, Valesca Dall’Alba, Anderson Garcez, Raquel Canuto, Maria Teresa Anselmo Olinto.

**Writing – review & editing:** Caio Wolff Ramos Baumstein, Maria Teresa Anselmo Olinto.

## References

[1] Wu Z McGoogan JM. Characteristics of and important lessons from the coronavirus disease 2019 (COVID-19) outbreak in china: summary of a report of 72 314 cases from the Chinese center for disease control and prevention. JAMA. 2020;323:1239–42. 32091533 10.1001/jama.2020.2648

[2] WHO. Timeline: WHO’s COVID-19 response. World Health Organization. 2021. https://www.who.int/emergencies/diseases/novel-coronavirus-2019/interactive-timeline. Accessed March 4, 2024.

[3] WHO. WHO COVID-19 dashboard. (2023, May 18). World Health Organization. 2023. https://covid19.who.int/. Accessed October 19, 2023.

[4] BRAZIL. Ministry of Health. Coronavírus Brasil. 2023. https://covid.saude.gov.br/. Accessed April 23, 2024.

[5] Olinto MTA Garcez A Brunelli G Olinto FA Fanton M Canuto R. Relationship between temperature and relative humidity on initial spread of COVID-19 cases and related deaths in Brazil. J Infect Dev Ctries. 2022;16:759–67. 35656945 10.3855/jidc.15324

[6] Oran DP Topol EJ. Prevalence of asymptomatic SARS-CoV-2 infection: a narrative review. Ann Intern Med. 2020;173:362–7. 32491919 10.7326/M20-3012 PMC7281624

[7] Stokes EK Zambrano LD Anderson KN. Coronavirus disease 2019 case surveillance: United States, January 22–May 30, 2020. MMWR Morb Mortal Wkly Rep. 2020;69:759–65. 32555134 10.15585/mmwr.mm6924e2 PMC7302472

[8] Berger JR. COVID-19 and the nervous system. J Neurovirol. 2020;26:143–8. 32447630 10.1007/s13365-020-00840-5 PMC7245181

[9] Ganesh A Reis IR Varma M Patry DG Cooke LJ. Neurological and head/eyes/ears/nose/throat manifestations of COVID-19: a systematic review and meta-analysis. Can J Neurol Sci. 2022;49:514–31. 34287109 10.1017/cjn.2021.180 PMC8460425

[10] Johnson BJ Salonen B O’Byrne TJ. Patient factors associated with COVID-19 loss of taste or smell patient factors in smell/taste loss COVID-19. Laryngoscope Investig Otolaryngol. 2022;7:1688–94. 10.1002/lio2.911 PMC9764767 36544937

[11] Shelton JF Shastri AJ Fletez-Brant K andMe C-T Aslibekyan S Auton A. The UGT2A1/UGT2A2 locus is associated with COVID-19-related loss of smell or taste. Nat Genet. 2022;54:121–4. 35039640 10.1038/s41588-021-00986-w

[12] Liu N Yang D Zhang T Sun J Fu J Li H. Systematic review and meta-analysis of olfactory and gustatory dysfunction in COVID-19. Int J Infect Dis. 2022;117:155–61. 35134561 10.1016/j.ijid.2022.02.004 PMC8817419

[13] Nouchi A Chastang J Miyara M. Prevalence of hyposmia and hypogeusia in 390 COVID-19 hospitalized patients and outpatients: a cross-sectional study. Eur J Clin Microbiol Infect Dis. 2021;40:691–7. 33033955 10.1007/s10096-020-04056-7 PMC7543958

[14] Lechien JR Chiesa-Estomba CM De Siati DR. Olfactory and gustatory dysfunctions as a clinical presentation of mild-to-moderate forms of the coronavirus disease (COVID-19): a multicenter European study. Eur Arch Otorhinolaryngol. 2020;277:2251–61. 32253535 10.1007/s00405-020-05965-1 PMC7134551

[15] da Costa KVT Carnauba ATL Rocha KW de Andrade KCL Ferreira SMS Menezes PDL. Olfactory and taste disorders in COVID-19: a systematic review. Braz. J. Otorhinolaryngol. 2020;86:781–92. 32580925 10.1016/j.bjorl.2020.05.008 PMC7280089

[16] Purja S Shin H Lee JY Kim E. Is loss of smell an early predictor of COVID-19 severity: a systematic review and meta-analysis. Arch Pharm Res. 2021;44:725–40. 34302637 10.1007/s12272-021-01344-4 PMC8302975

[17] Munoz-Rodriguez JR Gomez-Romero FJ Perez-Ortiz JM; COVID-19 SESCAM Network. Characteristics and risk factors associated with mortality in a multicenter Spanish cohort of patients with COVID-19 Pneumonia. Arch Bronconeumol. 2021;57:34–41. 34629641 10.1016/j.arbres.2021.02.021 PMC7939995

[18] Goshtasbi K Pang J Lehrich BM. Association between olfactory dysfunction and critical illness and mortality in COVID-19: a meta-analysis. Otolaryngol Head Neck Surg. 2022;166:388–92. 34030510 10.1177/01945998211017442

[19] Talavera B Garcia-Azorin D Martinez-Pias E. Anosmia is associated with lower in-hospital mortality in COVID-19. J Neurol Sci. 2020;419:117163. 33035870 10.1016/j.jns.2020.117163 PMC7527278

[20] Ninchritz-Becerra E Soriano-Reixach MM Mayo-Yanez M. Subjective evaluation of smell and taste dysfunction in patients with mild COVID-19 in Spain. Med Clin (Engl ed). 2021;156:61–4. 33521313 10.1016/j.medcle.2020.08.004 PMC7836722

[21] Borges RB Mancuso ACB Camey SA. Power and sample size for health researchers: a tool for calculating sample size and statistical power designed for health researchers. Clin Biomed Res. 2020;40:247–53.

[22] Gusmao PAOA Roveda JRC Leite ASM Leite AS Marinho CC. Changes in olfaction and taste in patients hospitalized for COVID-19 and their relationship to patient evolution during hospitalization. Braz. J. Otorhinolaryngol. 2022;88(Suppl 5):S75–82. 34876382 10.1016/j.bjorl.2021.11.002 PMC8610827

[23] Marcolino MS Ziegelmann PK Souza-Silva MVR; Brazilian COVID-19 Registry Investigators. Clinical characteristics and outcomes of patients hospitalized with COVID-19 in Brazil: Results from the Brazilian COVID-19 registry. Int J Infect Dis. 2021;107:300–10. 33444752 10.1016/j.ijid.2021.01.019 PMC7801187

[24] Amanat M Rezaei N Roozbeh M. Neurological manifestations as the predictors of severity and mortality in hospitalized individuals with COVID-19: a multicenter prospective clinical study. BMC Neurol. 2021;21:116. 33726699 10.1186/s12883-021-02152-5 PMC7960879

[25] Saussez S Lechien JR Hopkins C. Anosmia: an evolution of our understanding of its importance in COVID-19 and what questions remain to be answered. Eur Arch Otorhinolaryngol. 2021;278:2187–91. 32909060 10.1007/s00405-020-06285-0 PMC7480210

[26] Sheng WH Liu WD Wang JT Chang SY Chang SC. Dysosmia and dysgeusia in patients with COVID-19 in northern Taiwan. J Formos Med Assoc. 2021;120(1 Pt 2):311–7. 33139151 10.1016/j.jfma.2020.10.003 PMC7574720

[27] Mendonca CV Mendes Neto JA Suzuki FA Orth MS Machado Neto H Nacif SR. Olfactory dysfunction in COVID-19: a marker of good prognosis? Braz. J. Otorhinolaryngol. 2022;88:439–44. 33441276 10.1016/j.bjorl.2020.12.002 PMC7831803

[28] Bukhari AF Malas M Hanbazazah K Zawawi F. The incidence and impact of anosmia on daily life after COVID-19 infection: a cross-sectional study in a tertiary center in Saudi Arabia. Saudi Med J. 2022;43:1354–62. 36517057 10.15537/smj.2022.43.12.20220559 PMC9994517

[29] Mao L Jin H Wang M. Neurologic manifestations of hospitalized patients with coronavirus disease 2019 in Wuhan, China. JAMA Neurol. 2020;77:683–90. 32275288 10.1001/jamaneurol.2020.1127 PMC7149362

[30] Chawla J Y N Bakshi SS. Oral manifestations associated with COVID-19 disease: an observational cross sectional study. J Oral Biol Craniofac Res. 2022;12:279–83. 35340816 10.1016/j.jobcr.2022.03.008 PMC8934178

[31] Butowt R Bilinska K von Bartheld C. Why does the omicron variant largely spare olfactory function? Implications for the pathogenesis of anosmia in coronavirus disease 2019. J Infect Dis. 2022;226:1304–8. 35467743 10.1093/infdis/jiac113 PMC9129133

[32] Al-Rawi NH Sammouda AR AlRahin EA. Prevalence of anosmia or ageusia in patients with COVID-19 among United Arab Emirates Population. Int Dent J. 2022;72:249–56. 34226066 10.1016/j.identj.2021.05.006 PMC8141716

[33] Landis BN Hummel T. Measuring olfaction instead of asking: it is more than luxury! Eur Arch Otorhinolaryngol. 2020;277:1843–4. 31325033 10.1007/s00405-019-05565-8

